# Synthesis of Precision Gold Nanoparticles Using Turkevich Method

**DOI:** 10.14356/kona.2020011

**Published:** 2020-02-29

**Authors:** Jiaqi Dong, Paul L. Carpinone, Georgios Pyrgiotakis, Philip Demokritou, Brij M. Moudgil

**Affiliations:** 1 Center for Particulate and Surfactant Systems, Department of Materials Science and Engineering, University of Florida, USA; 2 HSPH-NIEHS Nanosafety Center, School of Public Health, Harvard University, USA

**Keywords:** gold nanoparticles, nanomaterials, Turkevich Method, synthesis, characterization

## Abstract

Gold nanoparticles (AuNPs) exhibit unique size-dependent physiochemical properties that make them attractive for a wide range of applications. However, the large-scale availability of precision AuNPs has been minimal. Not only must the required nanoparticles be of precise size and morphology, but they must also be of exceedingly narrow size distribution to yield accurate and reliable performance. The present study aims to synthesize precision AuNPs and to assess the advantages and limitations of the Turkevich method—one of the common chemical synthesis technique. Colloidal AuNPs from 15 nm to 50 nm in diameter were synthesized using the Turkevich method. The effect of the molar ratio of the reagent mixture (trisodium citrate to gold chloride), the scaled-up batch size, the initial gold chloride concentration, and the reaction temperature was studied. The morphology, optical property, surface chemistry, and chemical composition of AuNPs were thoroughly characterized. It was determined that the as-synthesized AuNPs between 15 nm and 30 nm exhibit well-defined size and shape, and narrow size distribution (*PDI* < 0.20). However, the AuNPs became more polydispersed and less spherical in shape as the particle size increased.

## 1. Introduction

Gold nanoparticle (AuNP) is one of the most exten- sively studied engineered nanomaterials (ENMs). The original work of AuNP synthesis can be traced back to 1940 when the formation of colloidal gold upon reacting gold chloride (HAuCl_4_) and trisodium citrate (Na_3_C_6_H_5_O_7_ or NaCt) was first reported (Hauser E.A. and Lynn J.E., 1940). The detailed work of Turkevich and his coworkers (Turkevich J. et al., 1951) has become one of the mile- stones of AuNPs synthesis. Since then, the synthesis method has been modified and improved (Frens G., 1973) for a diverse area of interests including the development of chemical sensors for water quality analysis using sur- face enhanced Raman spectroscopy (SERS) (Tian F. et al., 2014), surface-induced catalytic activities (Lopez N. et al., 2004; Xie W. et al., 2012), drug delivery in biological systems (Ghosh P. et al., 2008; Saallah S. and Lenggoro I.W., 2018) and nano-toxicology studies (Beltran-Huarac J. et al., 2018; Somasundaran P. et al., 2010; Grobmyer S.R. and Moudgil B.M., 2010; Pyrgiotakis G. et al., 2018). The reason that AuNPs are attractive to a wide variety of applications is because of the surface plasmon resonance (SPR), a size- and shape-dependent property (Haiss W. et al., 2007) and their biocompatibility (Sondi I. and Salopek-Sondi B., 2004; Zhang X., 2015).

Despite all the unique properties of AuNPs, the precision control of the particle size and size distribution presents a major challenge in this field often due to the fact of batch-to-batch variation in a local temperature gradient, the efficiency of reagent mixing and the resulting local concentration gradient (Kimling J. et al., 2006). For this reason, the availability of high precision AuNPs is rather limited and the price could easily go above $10,000 per gram. So far, only a few studies have discussed the method and effect of scaled-up AuNP synthesis. This study discusses the detailed procedure to synthesize precision AuNPs with the mean particle size between 15 nm and 30 nm, polydispersity index (*PDI*) less than 0.20, and scale up the batch reactor size to 1.5 L.

## 2. Materials and methods

### 2.1 Materials

Gold (III) chloride hydrate (99.995 % trace metals ba- sis) and trisodium citrate dehydrate (ACS reagent, ≥ 99.0 %) were purchased from Sigma-Aldrich and used without further purification. Hydrochloric acid (certified ACS Plus) and nitric acid (certified ACS Plus) were purchased from Fisher Scientific. Deionized water (18.2 MΩ) was used for all procedures.

### 2.2 Synthesis of AuNPs

All flasks used as reaction vessels were cleaned using freshly prepared aqua regia. Aqua regia was prepared using concentrated hydrochloride acid and concentrated nitric acid with the volume ratio of 4:1 respectively.

In a typical AuNP synthesis, 50 ml of 0.25 mM gold chloride (HAuCl_4_) solution was prepared in a flask. Independently, 34.0 mM (1.0 wt.%) trisodium citrate (NaCt) solution was prepared. The flask containing HAuCl_4_ solution was heated using a hotplate with constant and vigor- ous stirring. In order to avoid contamination and evaporation of the solvent during the synthesis, a dispos- able Petri dish was used to cover the flask. After the HAuCl_4_ solution reached the boiling point under ambient pressure, a specific volume of NaCt solution was rapidly injected into the HAuCl_4_ solution. The molar ratio (*MR*) of NaCt to HAuCl_4_ was the primary factor controlled to achieve the desired particle size (Frens G., 1973). The synthesis was complete when the color of the suspension no longer changed. Typically, the reaction took 2–5 min depending on the *MR*. The sample was cooled naturally to room temperature.

In a scaled-up AuNP synthesis, the volume of the HAuCl_4_ and NaCt solution were proportionally increased. The HAuCl_4_ solution was heated and vigorously stirred. The injection of a larger volume of NaCt solution was done using multiple disposable syringes to ensure fast and efficient mixing.

### 2.3 Characterization of AuNPs

The optical property and morphology of AuNPs were characterized using the following techniques for the pur- pose of general screening.

#### UV-visible spectroscopy.

UV-visible spectra were acquired using an Ocean Optics USB2000 + XR1-ES UV/ Visible spectrometer with DH-mini light source.

#### Dynamic light scattering (DLS).

The particle size, size distribution, and polydispersity index (*PDI* ) were obtained using a Malvern Zetasizer Ultra (Malvern Panalytical Ltd). All measurements were made at room temperature (25 °C).

#### Transmission electron microscopy (TEM).

Micrographs of AuNPs were acquired using FEI TECNAI F20 S/TEM. The particle size and size distribution of each sample were obtained by image analysis using Image J.

A more in-depth analysis of a representative sample of AuNPs was carried out at HSPH-NIEHS Nanosafety Center. In addition to particle morphology, the surface composition and chemical composition were analyzed in detail.

#### X-ray photoelectron spectroscopy (XPS).

The surface composition of AuNPs was analyzed using Thermo Scientific K-Alpha XPS system. The sample was prepared by repetitive spraying and drying AuNP suspension on Si wafer until 1 mg of AuNPs in total was deposited. Avan- tage™ Software (Thermo Scientific, Waltham, MA) was used to calculate the elemental composition.

#### Fourier transform infrared spectroscopy (FT-IR).

The Perkin Spectrum One ATR was used to obtain the infrared spectrum of a representative sample of AuNPs. The spectrum was analyzed manually with all peaks identified based on the NIST FT-IR database.

#### Inductively coupled plasma mass spectrometry (ICP-MS).

The elemental composition of a representative sample of AuNPs was analyzed using Thermo-Finnigan Element 2. The protocol (Herner J.D. et al., 2006) of sam- ple preparation and composition evaluation was followed.

## 3. Characterization

### 3.1 UV-visible spectrum

**Fig. 1:**
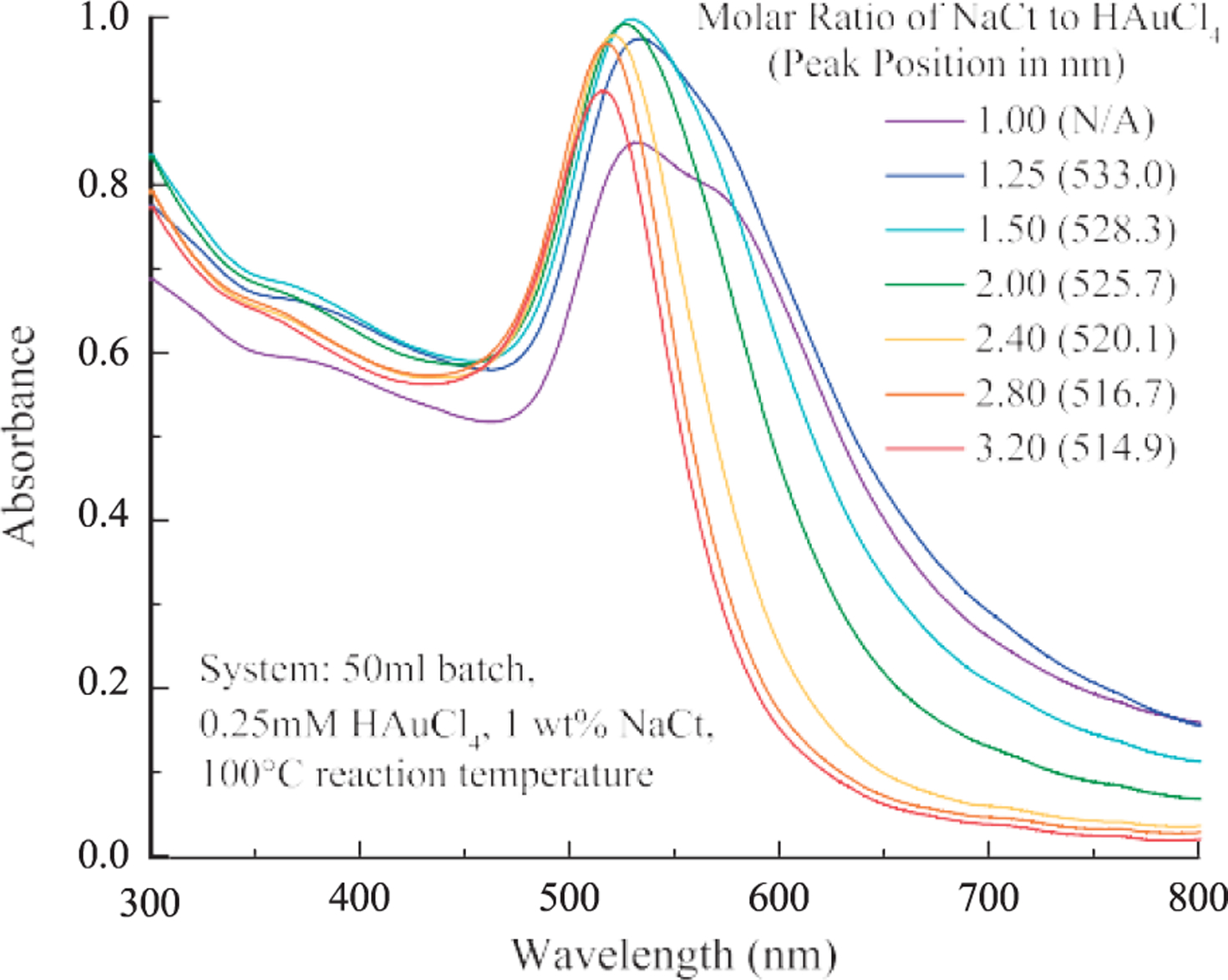
The SPR peak position of AuNPs shifted to shorter wavelength and the peak width decreased as the molar ratio increased from 1.00 to 3.20.

The blue shift of the SPR peak center and the reduction of peak width (Fig. 1) were indicative of the decrease in particle size and the polydispersity, respectively, as the molar ratio increases. A shoulder on the spectrum was observed for the samples with the molar ratio below 2.00, which indicated the AuNPs could be anisotropic in shape (Kimling J. et al., 2006) or a certain degree of agglomera- tion. Literature suggested that the reason was probably due to insufficient control of the nucleation and growth events and/or insufficient particle stabilizing when less NaCt was used in the reaction (Wuithschick M. et al., 2015). In contrast, the AuNP synthesized using the molar ratio between 2.00 and 3.20 exhibited narrower peak width.

### 3.2 Dynamic light scattering

**Fig. 2:**
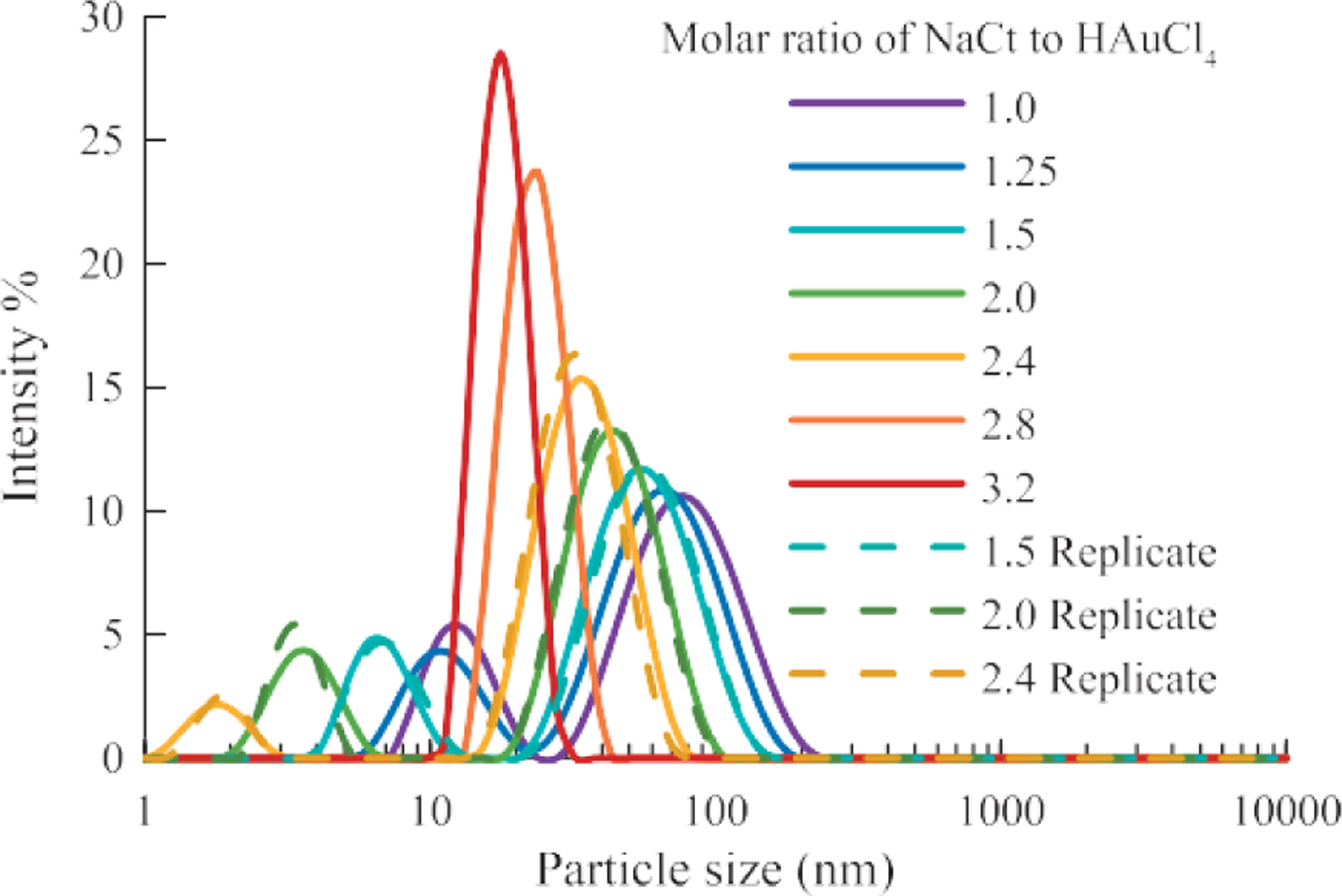
The particle size of AuNPs increased as the molar ratio decreased. A bimodal distribution was observed for AuNP samples with *MR* ≤ 2.40. (System: 50 ml batch size, 0.25 ml HAuCl_4_, 1 wt% NaCt, 100 °C reaction temperature)

**Fig. 3:**
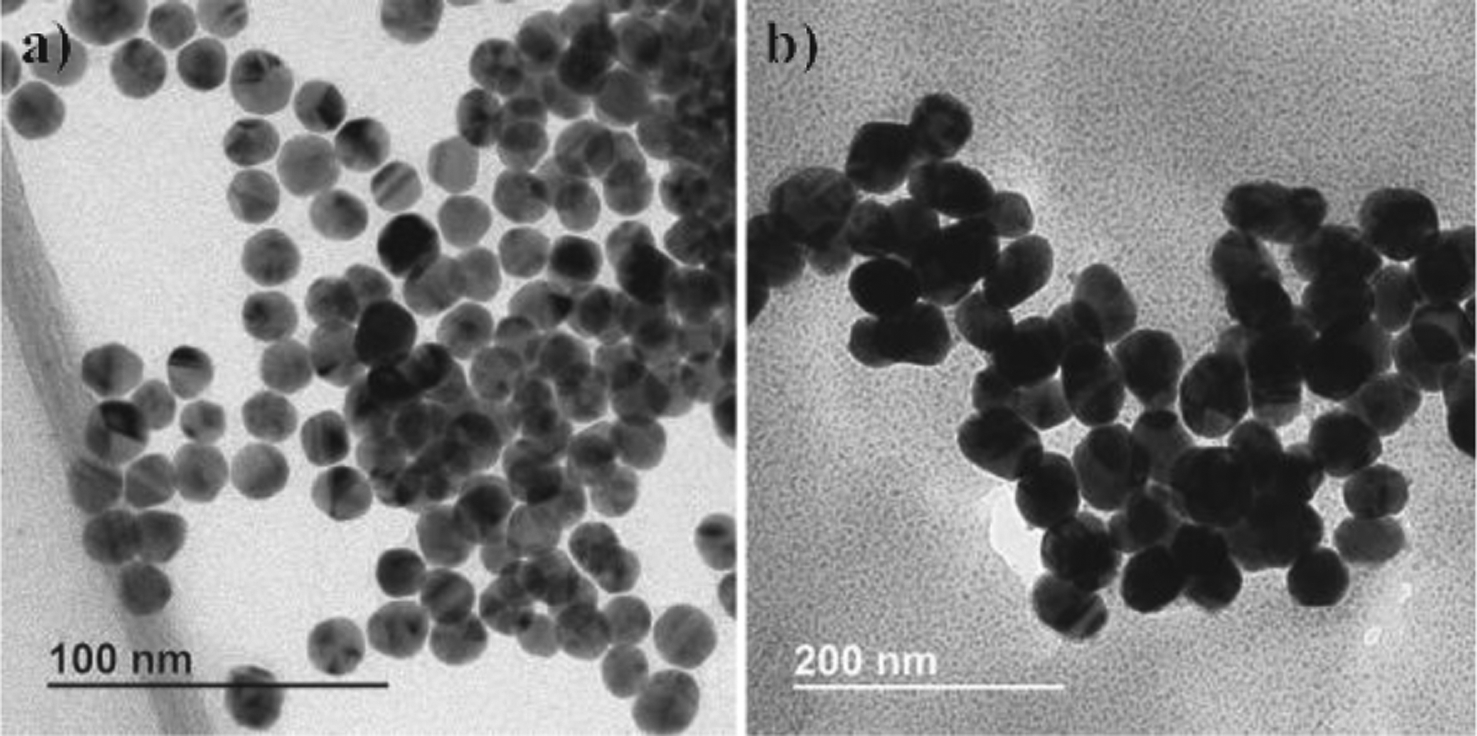
TEM images of as-synthesized AuNPs **a)** *MR* = 2.80, mean size = 15 nm, batch size = 1.5 L; **b)** *MR* = 1.50, mean size = 50 nm, batch size = 1.5 L.

DLS measurements (Fig. 2) showed that relatively uniform (*PDI* < 0.20) AuNPs between 15 and 30 nm were synthesized using the Turkevich method. The *PDI* in- creases as the particle size becomes larger. As the *MR* de- creased below 2.4, bimodal particle size distribution was observed with sub-10 nm particles present in addition to the major peak. The presence of the sub-10 nm was confirmed by TEM images in Fig. 3b & 11.

### 3.3 Transmission electron microscopy

TEM image analysis confirmed the DLS measurement of particle size, as shown in Fig. 3 with image analysis of the AuNP samples in Table 1. Also, slightly elongated particles were ob- served for the 50 nm sample.

### 3.4 XPS, FI-IR, and ICP-MS

**Fig. 4:**
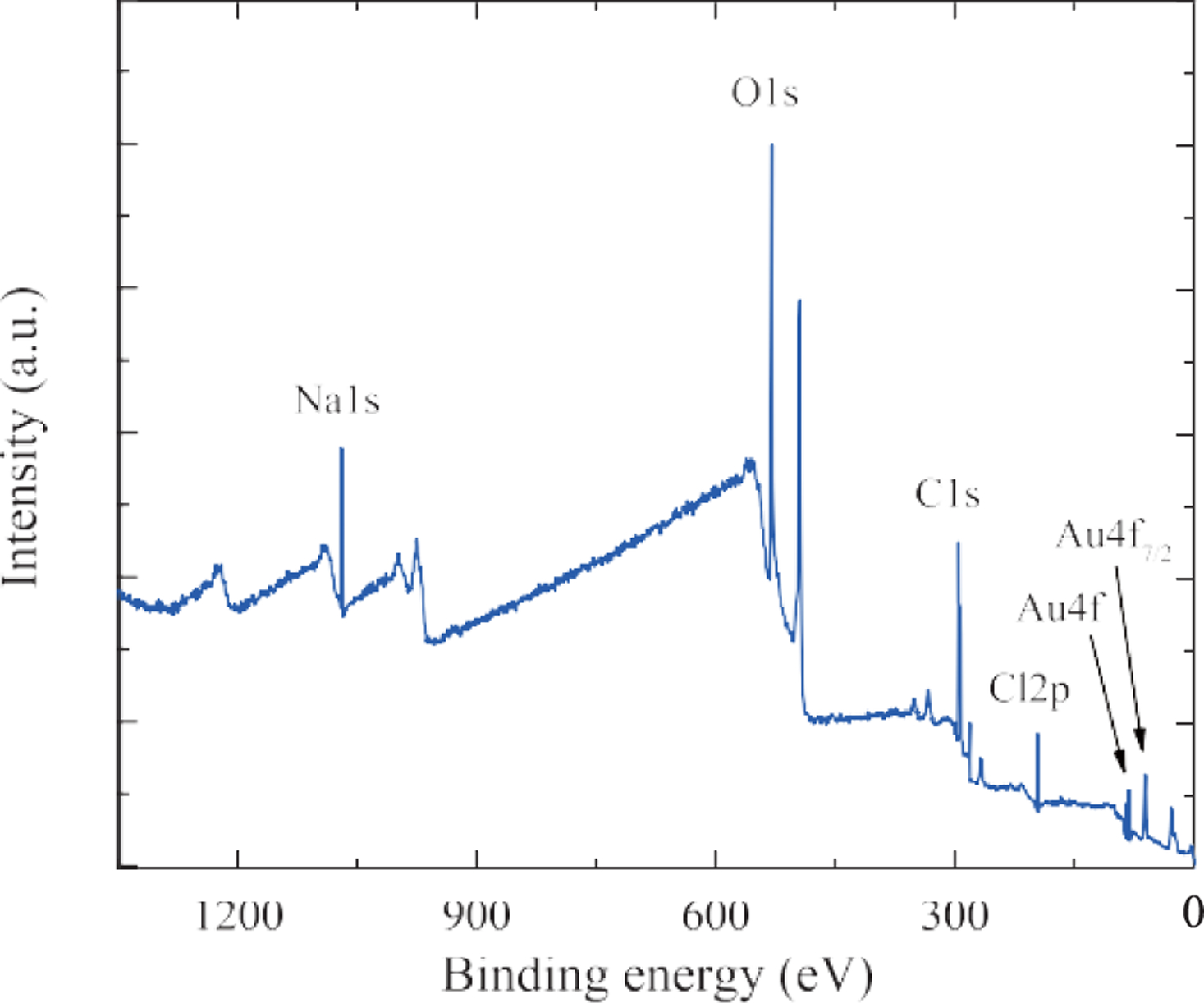
XPS data of the 15 nm AuNPs sample.

**Fig. 5:**
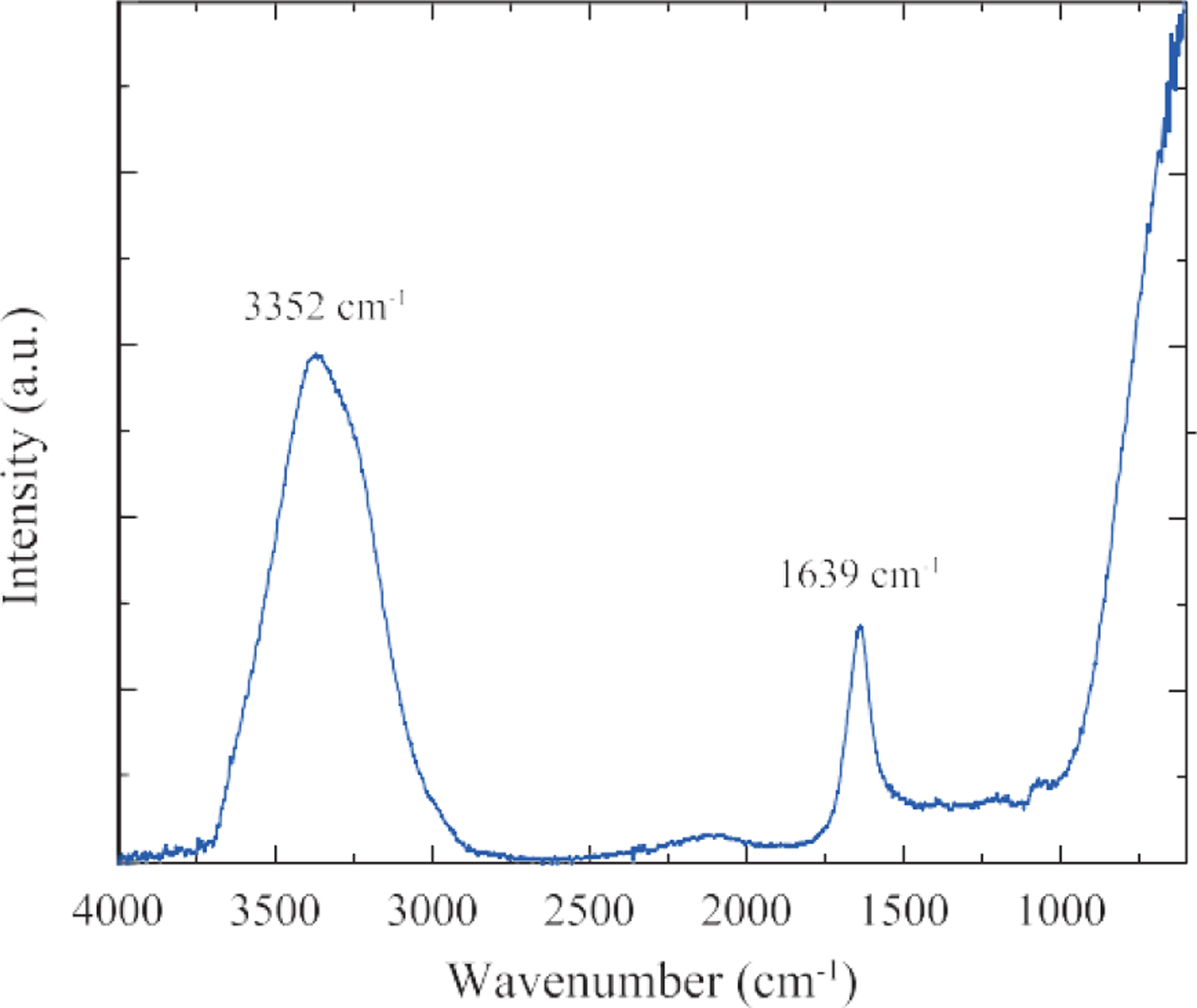
FT-IR data of the 15 nm AuNPs sample.

The XPS (Fig. 4) and FT-IR (Fig. 5) data, in Table 2 and Table 3, respectively, showed the presence of carbon, oxygen, sodium and chlorine elements on the surface of AuNPs, which was typical for AuNPs prepared using the Turkevich method.

Finally, the chemical composition of the colloidal AuNPs sample was verified by ICP-MS. The results sum- marized in Table 4 indicated that the sample was highly pure (> 99.9 %).

## 4. Results and discussion

### 4.1 Effect of molar ratio

**Fig. 6:**
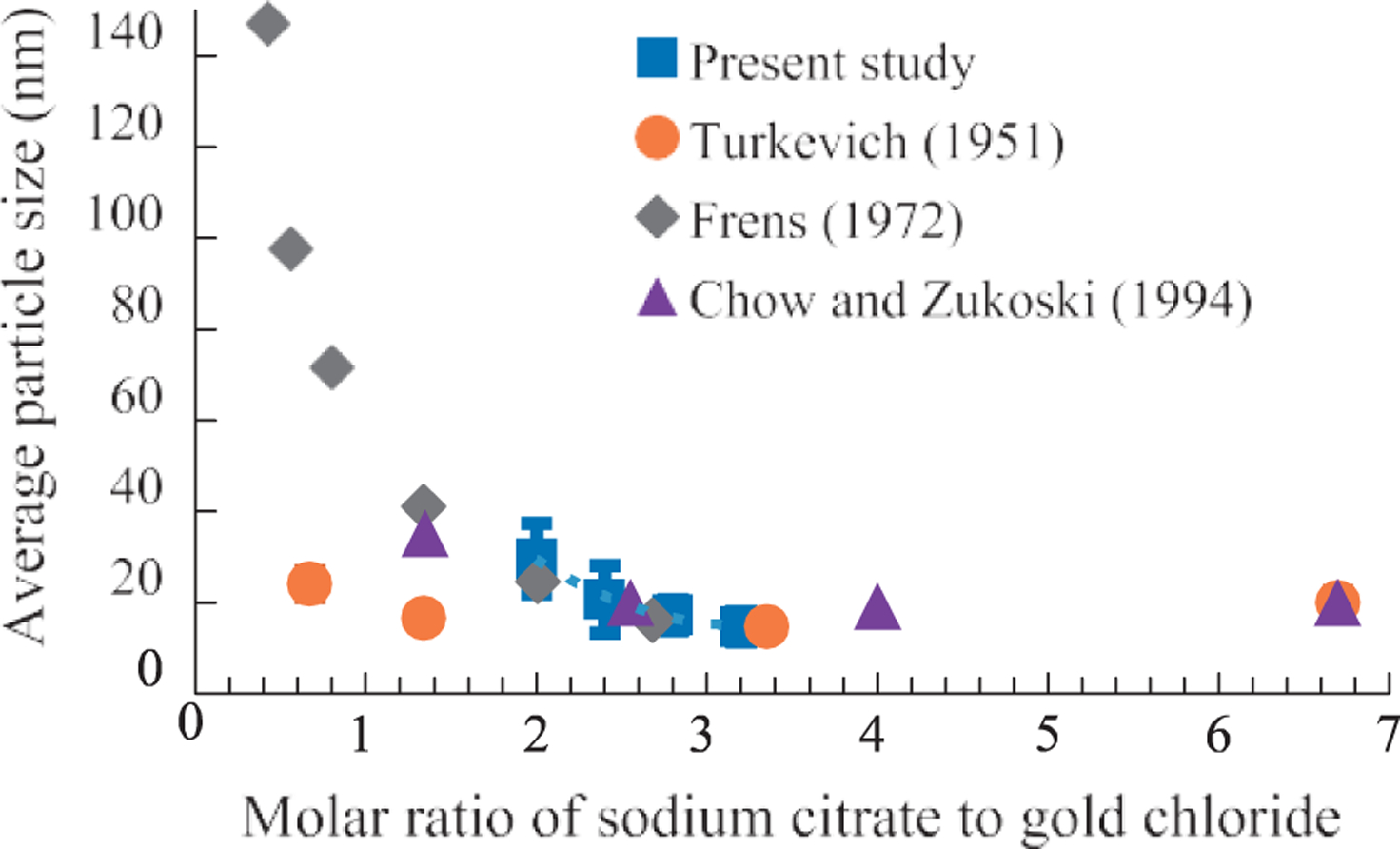
Control of particle size by changing the molar ratio of NaCt to HAuCl_4_ with the Turkevich method.

One of the advantages of the Turkevich method is the ability to control the AuNP size by changing the molar ratio of NaCt to HAuCl_4_. Typically, AuNPs between 10 nm and 150 nm can be synthesized by decreasing the molar ratio (*MR*) from 4 to 0.5 as shown in Fig. 6 (Chow M.K. and Zukoski C.F., 1994; Frens G., 1973; Turkevich J. et al., 1951). Further decrease in the *MR* would result in an incomplete reduction of HAuCl_4_ to AuNPs.

According to the proposed mechanism (Polte J., 2015; Polte J. et al., 2010), large numbers of seed particles (~2 nm in radius) were formed as the supersaturation of the gold atoms increased rapidly. If there was sufficient ci- trate in the solution (*MR* > 3) the seed particles would be stabilized, and the AuNPs growth process and their final particle size would be approximately the same regardless of the molar excess (Wuithschick M. et al., 2015). As the *MR* decreases (*MR* < 3) there would be less citrate available to stabilize the seed particles. Consequently, aggregation of the seed particles would occur, which results in fewer particles, larger final particle sizes, and less spherical particle shape. A recent study provides evidence of coalescence of 2 nm citrate-capped AuNPs in solution (Zhu C. et al., 2018), which supports the mechanism proposed by Polte et al. The aggregation of seeds would stop after the particle concentration was significantly reduced. The presence of aggregates could induce a self-catalytic effect which promotes the reduction of Au^3+^ on the aggregate surface itself. Eventually, the growth stopped when all precursor is consumed in the reaction.

### 4.2 Effect of batch size

**Fig. 7:**
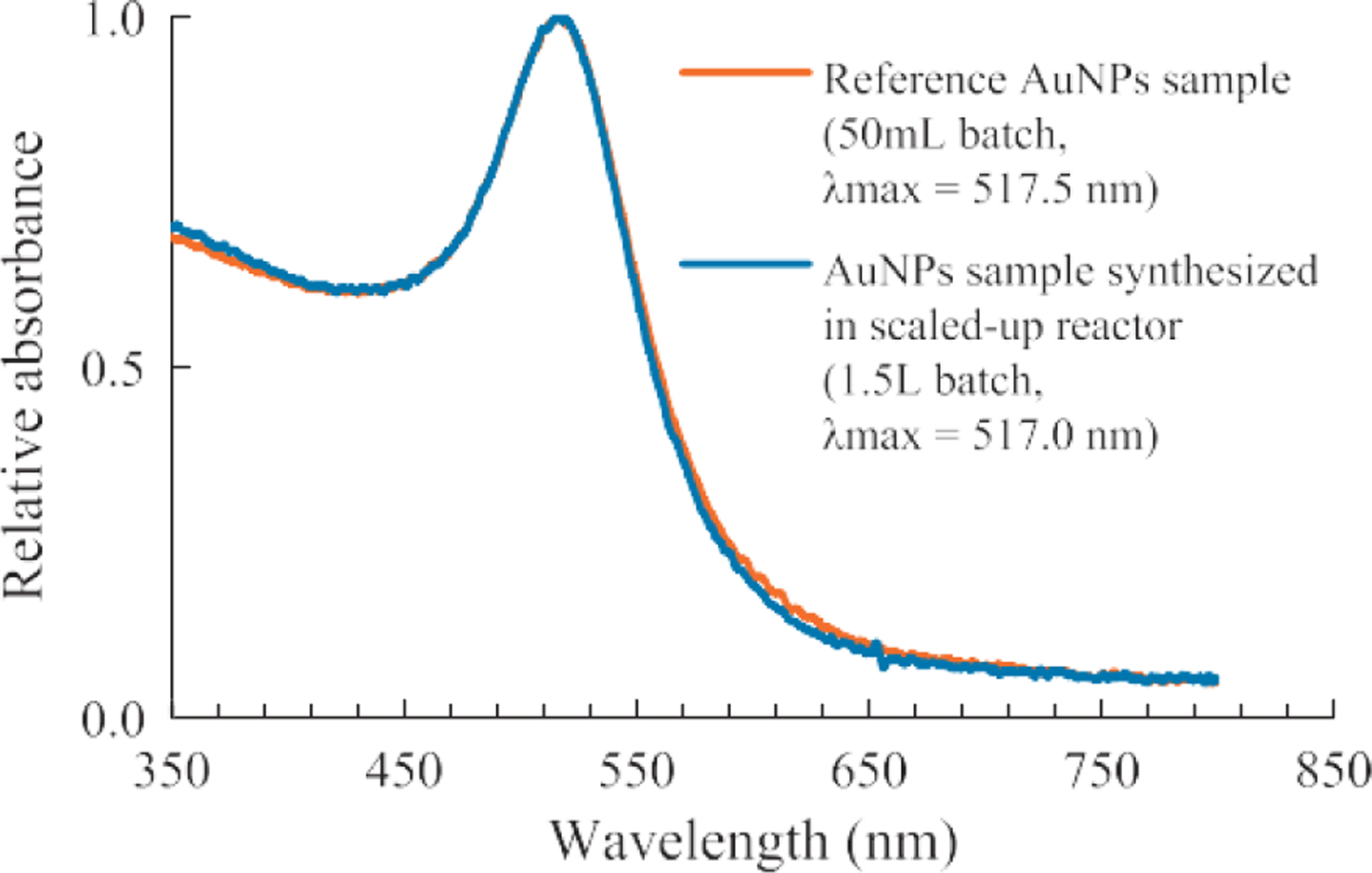
Batch size had minimum effect on the UV-visible spectrum of AuNPs synthesized (System: 0.25 ml HAuCl_4_, 1 wt% NaCt, *MR* = 2.80, 100 °C reaction temperature).

**Fig. 8:**
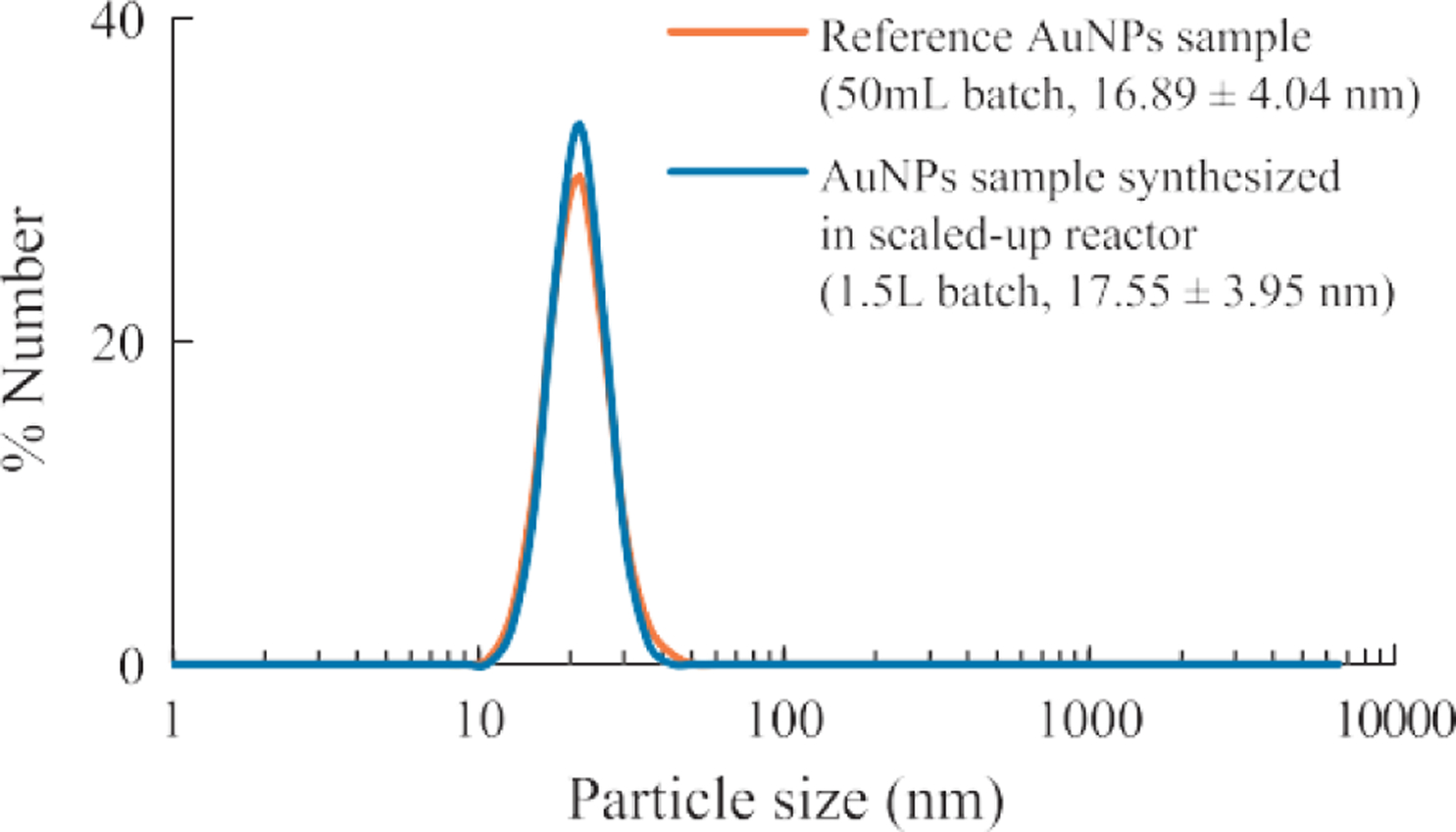
Batch size had a minimum effect on the particle size and size distribution of AuNPs synthesized under the given conditions. (System: 0.25 ml HAuCl_4_, 1 wt% NaCt, *MR* = 2.80, 100 °C reaction temperature).

The scaled-up AuNP synthesis was done by increasing the size of the reaction vessel as well as by proportionally increasing the reagent volumes. The effect of the batch size on the AuNPs was studied using UV-visible spectrum and the DLS measurement. In the experiment, AuNP synthesized in 50 mL batch vs. 1.5 L batch were compared. The results showed that there was minimal difference in the optical properties (Fig. 7) as well as the particle size, size distribution (Fig. 8) within the tested batch sizes.

### 4.3 Effect of initial HAuCl_4_ concentration

**Fig. 9:**
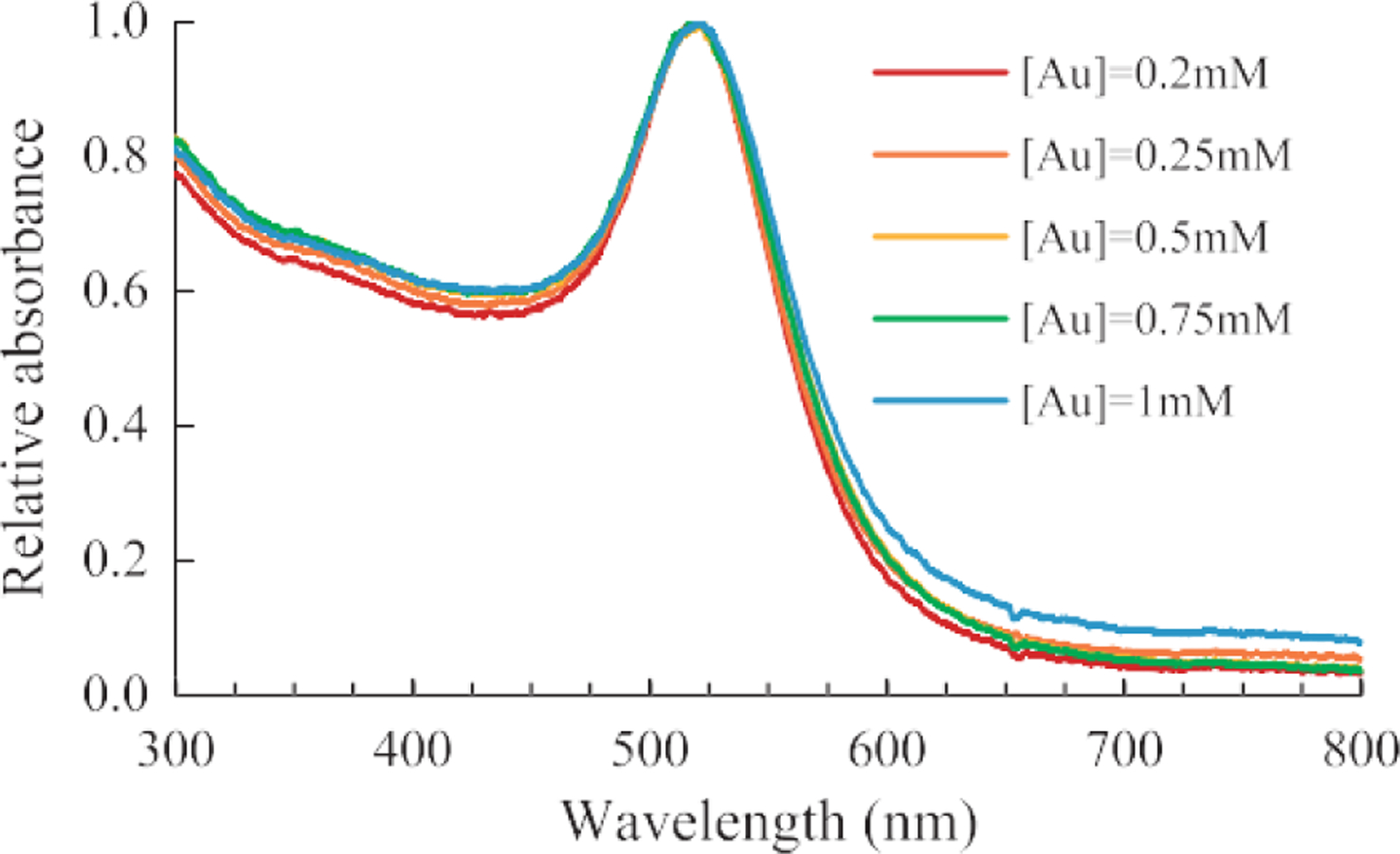
UV-visible spectra of AuNPs samples synthesized with specific initial gold chloride concentration. (System: 50 ml batch size, *MR* = 2.5, 100 °C reaction temperature)

The effects of initial HAuCl_4_ concentration on AuNP size and distribution was investigated. In the experiment, the molar ratio of NaCt to HAuCl_4_ was held constant at 2.5. AuNP was synthesized under initial HAuCl_4_ concentrations between 0.2 mM and 1 mM. The SPR peak positions of the spectra in Fig. 9 stayed relatively constant as the concentration increased, which suggested that the AuNPs particle sizes were not significantly impacted by the initial concentration under the test conditions. However, the peak breadth increased slightly as the initial concentration increased, which indicated that AuNPs became more polydispersed. The results agreed with the literature (Zabetakis K. et al., 2012) that the polydispersity was reduced when the initial HAuCl_4_ concentration was low (< 0.8 mM). However, the AuNP size and size distribution would be impacted if the reagent concentration was sufficiently high due to increased ionic strength and reduced electrostatic repulsion between AuNPs, thus reduced colloidal stability.

### 4.4 Effect of reaction temperature

Turkevich showed that a decrease in temperature would result in a decrease in particle size (Turkevich J. et al., 1951). At relatively lower temperatures (< 90 °C), the overall reduction rate and thus the nucleation rate was lower, so that fewer seed particles would be initially produced as compared to a higher temperature. Because the concentration of HAuCl_4_ was initially the same, the final particle size will be larger if there were fewer seed particles assuming equivalent conversion rate at all temperatures tested.

**Fig. 10:**
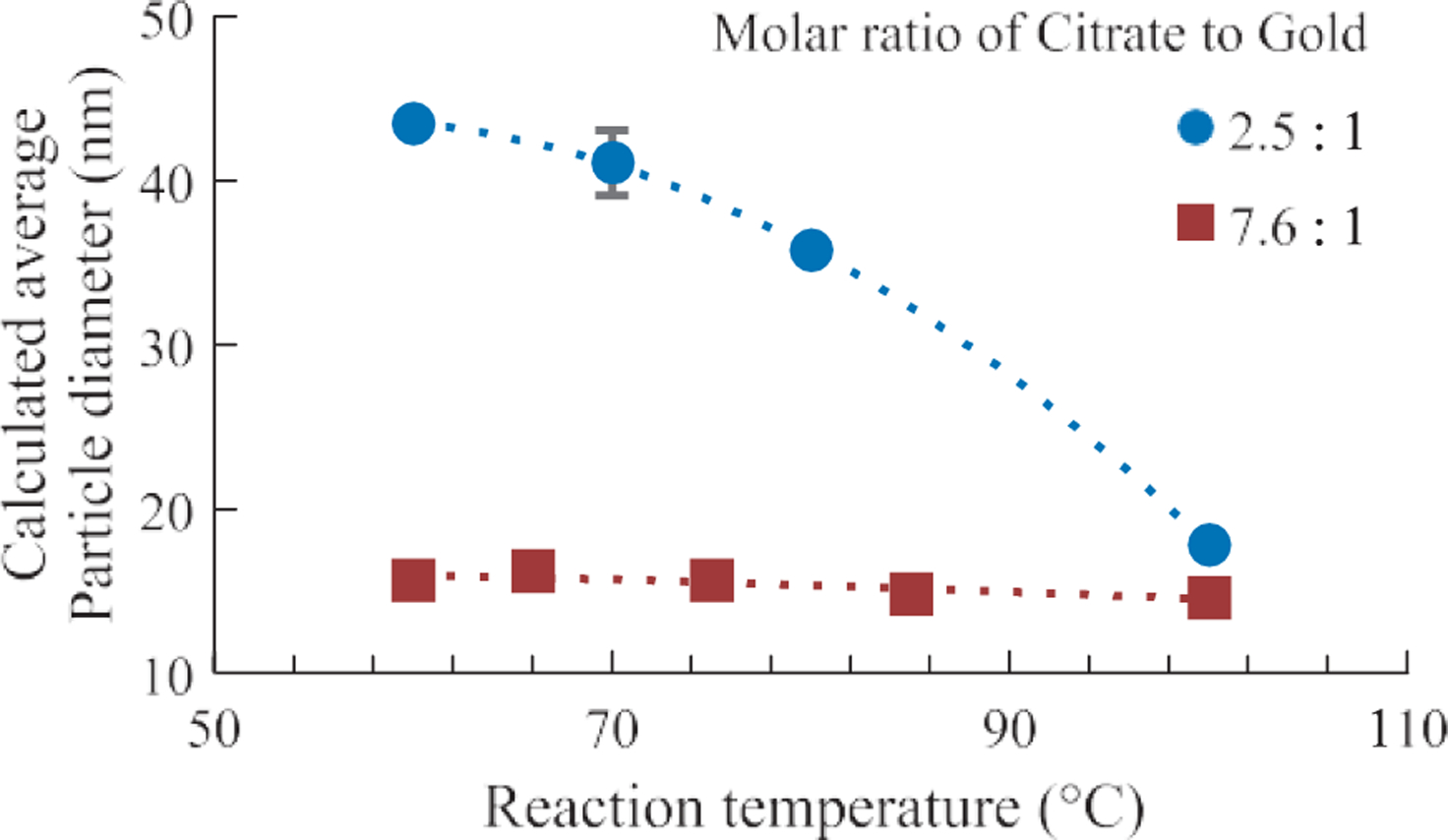
The change in AuNP diameter as a function of the reaction temperature in Turkevich synthesis. The diameter was calculated using the method described by Haiss et al. (Haiss W. et al., 2007)

In the present study, the effects of temperature was tested at two different molar ratios of citrate to gold as shown in Fig. 10. At the molar ratio of 2.5 to 1, the particle size decreased with increasing reaction temperature. These results can be explained by the change in kinetics described above. However, at a higher molar ratio, e.g. 7.6:1, no measurable change in the particle size was observed.

### 4.5 Other factors in the Turkevich method

In recent years, the Turkevich method has been further investigated in order to achieve more precise control over the particle size and size distribution by adjusting the reaction conditions. Literature reports suggest that there are additional critical parameters in Turkevich method, such as pH (Li C. et al., 2011; Schulz F. et al., 2014), the order of reagent addition (Ojea-Jiménez I. et al., 2011; Schulz F. et al., 2014; Sivaraman S.K. et al., 2011) and the latent heat of the reaction (Ding W. et al., 2015).

The solution pH could play a critical role in the AuNPs formation. The precursor HAuCl_4_ exists as different complexes [AuCl_4–x_(OH_x_)]^–^ depending on the solution pH (Wuithschick M. et al., 2015). As the pH of the solution increases, the Cl^–^ anions would be exchanged with OH^–^. The HAuCl_4_ complex becomes less reactive as more Cl^–^ anions are exchanged. The percentage of each complex would affect the overall reduction rate. In the Turkevich synthesis, the solution pH was determined by the initial concentration of HAuCl_4._ In addition, the reductant NaCt also served as a pH buffer. When NaCt was initially added to the HAuCl_4_ solution, the pH quickly increased, thereby transforming the reactive HAuCl_4_ complex into a less reactive complex. During this process, the nucleation was induced rapidly by the reactive HAuCl_4_ complex. The nucleation process subsequently would slow down and stop due to the transformation of the reactive gold complex into hydroxylated gold complexes that were less reactive (Wuithschick M. et al., 2015).

Another critical factor reported in the Turkevich method was the order of reagent addition. In the standard synthesis protocol, NaCt was injected into a boiling HAuCl_4_ solution. In the literature, it was reported that by injecting HAuCl_4_ into boiling NaCt a smaller final particle size can be produced with a narrower size distribution (Ojea-Jiménez I. et al., 2011; Sivaraman S.K. et al., 2011). In the conventional synthesis, citrate was rapidly injected, thereby not allowing it to convert into other species, whereas if the order of addition was reversed, the citrate solution was brought to boiling point first, allowing it to transform into different species in the heated solution before reacting with HAuCl_4_. Previous researchers believed that dicarboxyacetone, a product of citrate thermal oxidation, could play a significant role in Turkevich synthesis (Turkevich J. et al., 1951; Wuithschick M. et al., 2015). The decrease in particle size could be attributed to the increase in dicarboxyacetone concentration due to thermal oxidation of NaCt during the heating process. Further studies are needed to determine the exact mechanism of citrate oxidation and the role of dicarboxyacetone in the AuNPs formation.

Last but not the least, the effect of latent heat of the boiling HAuCl_4_ solution was also believed to be a factor that affects the formation of AuNPs in Turkevich synthesis (Ding W. et al., 2015). It was reported that an increase of the latent heat could lead to a reduction of approximately 3 nm in final particle size. Ding and co-workers believed that the decrease in the final particle size was due to increased nucleation and growth rate as more heat was provided during the reaction.

### 4.6 Limitations of the Turkevich method

**Fig. 11:**
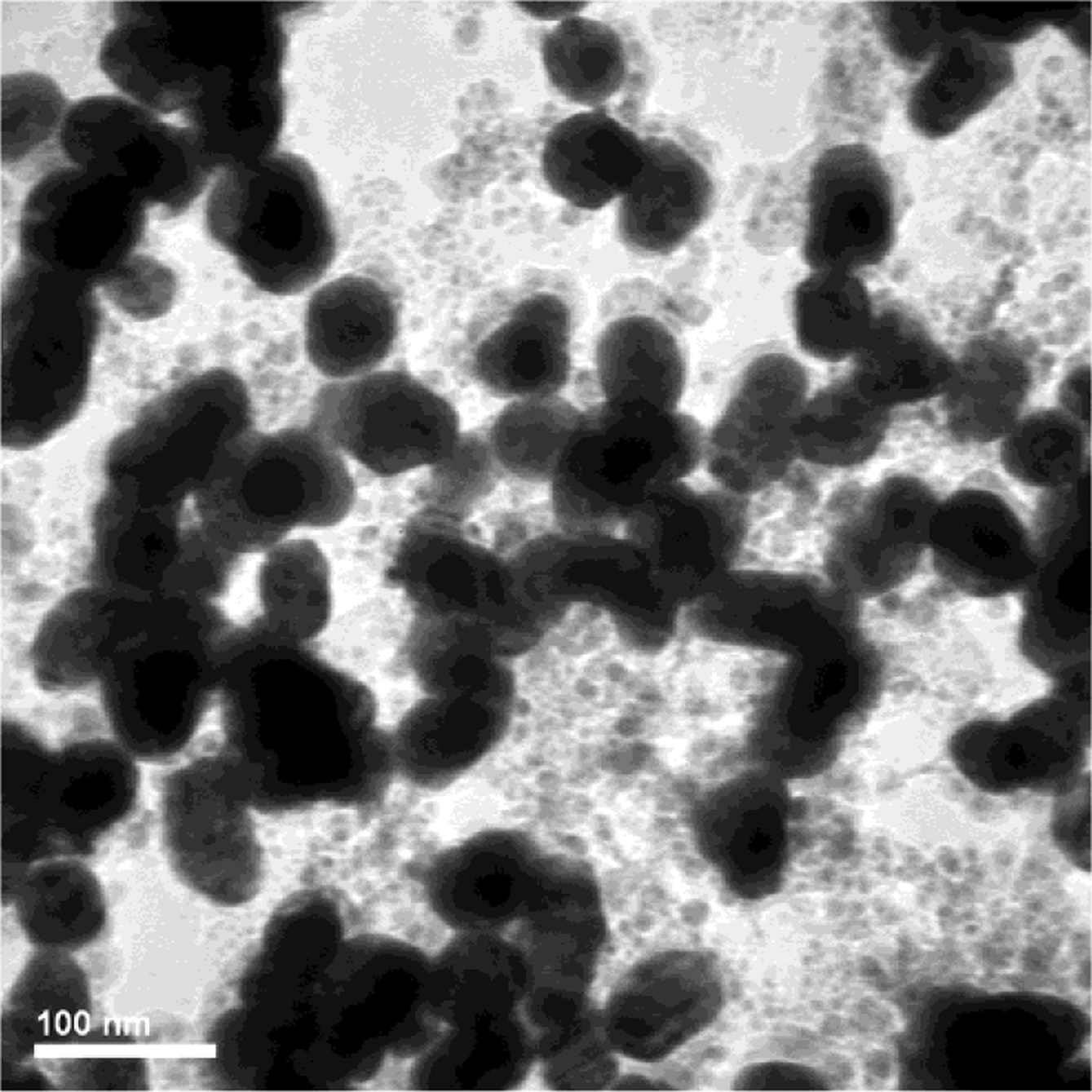
Sub-10 nm AuNPs (5–8 nm) was observed in the as-synthesized 50 nm AuNP sample with the *MR* = 1.5.

The Turkevich method is a relatively simple and reproducible technique for the synthesis of spherical particles between 10 nm to 30 nm. However, the particles become less spherical, the size distribution becomes broader, and the results were less reproducible for the synthesis of AuNP above 30 nm size. Bimodal particle size distributions were observed for AuNP samples synthesized with *MR* ≤ 2.4 as shown in Fig. 2. The presence of sub-10 nm particles can be observed in the AuNPs sample (*MR* = 1.5) by TEM as shown in Fig. 11. It was hypothesized that the presence of sub-10 nm AuNPs could be attributed to incomplete Oswald’s ripening which happened when NaCt concentration was relatively low in the reaction to stabilize all AuNPs. However, it was not entirely understood and warrants further study.

## 5. Conclusions

Gold nanoparticle (Au NP) synthesis using the Turkevich method was revisited. The effects of molar ratio, batch size, reagent concentration, and the temperature were investigated in this study. The particle size, size distribution, morphology of the AuNPs were characterized in detail, and the results agreed with the values reported in the literature. The AuNPs size was tuned from 15 nm to 50 nm by decreasing the molar ratio of NaCt to HAuCl_4_ from 2.8 to 1.5. However, the AuNPs became more polydispersed and less spherical as the molar ratio decreased. The batch synthesis was scaled up to 1.5 L and the as-synthesized AuNPs exhibited identical optical property and morphology as the AuNPs synthesized in 50 ml batches. At a constant molar ratio, the initial concentration of HAuCl_4_ had minimal effect on the final particle size and size distribution within the range tested. The particle size increased with decreasing reaction temperature at the molar ratio of 2.5. However, there was no significant effect of temperature on the particle size at the molar ratio of 7.6.

Other potentially important factors reported in the literature include the solution pH, the order of reagent addition, and the latent heat. These factors were not examined in the present study, and need further investigation. Overall, the Turkevich method was found to be reliable for producing precision gold nanoparticles (*PDI* < 0.20) between 15 nm and 30 nm.

## Figures and Tables

**Table 1:** TEM image analysis of the representative AuNP samples.

Sample	15 nm AuNP	50 nm AuNP
Count (#)	124	103
Mean Diameter ± SD (nm)	16.99 ± 1.41	51.6 ± 5.16
Media Diameter (nm)	17.00	51.83
Aspect Ratio ± SD	1.10 ± 0.07	1.22 ± 0.14
Roundness ± SD	0.91 ± 0.05	0.83 ± 0.09

**Table 2:** XPS data of the 15 nm AuNP sample.

Element	Peak	Atomic Percentage
Gold	Au4f	0.51
Oxygen	O1s	38.72
Carbon	C1s	45.39
Chloride	Cl2p	3.19
Sodium	Na1s	12.20

**Table 3:** FT-IR data of the 15 nm AuNPs sample.

Peak (cm^−1^)	Correspondence
3352 cm^−1^	Water
1639 cm^−1^	Water, Citrate

**Table 4:** Summary of chemical composition analysis of the 15 nm AuNPs.

Inductively Coupled Plasma Mass Spectrometry	Purity of AuNP	99.99 ± 0.31 %
	Concentration of AuNP	51.296 ± 1.657 μg/ml
